# Circulating growth differentiation factor 15 levels and apolipoprotein B to apolipoprotein A1 ratio in coronary artery disease patients with type 2 diabetes mellitus

**DOI:** 10.1186/s12944-022-01667-1

**Published:** 2022-07-16

**Authors:** Yufeng Mei, Zhiming Zhao, Yongnan Lyu, Yan Li

**Affiliations:** 1 Department of Clinical Laboratory, Renmin Hospital of Wuhan University, Wuhan, Hubei Province 430060 People’s Republic of China; 2 Department of Geratology, Renmin Hospital of Wuhan University, Wuhan, Hubei Province 430060 People’s Republic of China; 3 Department of Cardiology, Renmin Hospital of Wuhan University, Wuhan, Hubei Province 430060 People’s Republic of China

**Keywords:** T2DM, GDF-15, ApoB/ApoA1 ratio, CAD

## Abstract

**Background:**

Clinical investigations have found that there was a close association between T2DM and adverse cardiovascular events, with possible mechanisms included inflammation, apoptosis, and lipid metabolism disorders. High serum GDF-15 concentration and the apolipoprotein B/apolipoprotein A1 ratio (ApoB/ApoA1) are involved in the above-mentioned mechanisms and are thought to be related to the occurrence of adverse cardiovascular events. However, it remains unclear whether circulating GDF-15 levels and the ApoB/ApoA1 ratio are related to T2DM patients with CAD.

**Methods:**

T2DM patients with or without CAD were eligible for this study. According to the inclusion and exclusion criteria, 502 T2DM patients were enrolled between January 2021 and December 2021 and were then divided into T2DM group (*n* = 249) and CAD group (*n* = 253). The ApoB, ApoA1 and GDF-15 concentrations were measured at hospital admission and the ApoB/ApoA1 ratio was then calculated.

**Results:**

Compared with T2DM group, serum GDF-15 levels and ApoB/ApoA1 ratio increased in CAD group. Furthermore, a positive relationship between the occurrence of CAD in diabetic population and circulating GDF-15 concentrations and ApoB/ApoA1 ratio was observed in logistic regression analysis (*p* < 0.01). Restrictive cubic spline analysis after adjusted for multiple risky variables showed that serum GDF-15 or ApoB/ApoA1 ratio correlated positively with CAD.

**Conclusions:**

Circulating GDF-15 levels and serum ApoB/ApoA1 ratio vary in CAD group and T2DM group. ApoB/ApoA1 and GDF-15 may be helpful for predicting the occurrence of CAD in patients with T2DM.

**Supplementary Information:**

The online version contains supplementary material available at 10.1186/s12944-022-01667-1.

## Introduction

Type 2 diabetes mellitus (T2DM) is the most common form of diabetes. It is estimated that patients with diabetes mellitus in the world will reach 783.2 million in 2045, of which T2DM will account for more than 90% [1]. Despite the advent of insulin and exercise and dietary management largely controlling the progression of T2DM, comorbidities associated with the disorder, especially coronary artery disease (CAD), are still an important cause of death [2, 3]. Researchers have therefore turned their attention to novel biomarkers for predicting CAD in T2DM patients [4, 5].

GDF-15 is one of the new metrics and many recent studies have showed that its levels correlate closely with pancreatic β-cell apoptosis, vascular endothelial cell and adipocyte inflammatory damage and oxidative stress [6–11]. Possible mechanisms for this association are: 1) mediating vascular endothelial damage and atherosclerotic plaque formation via the TGFβRII pathway [12], 2) regulating apoptosis and IL − 6-related vascular damage [10], 3) combination with oxidized low-density lipoprotein(ox-LDL) to ruin macrophage autophagy then interfere with macrophage lipid homeostasis [11], and 4) maintaining the activity of the PI3K/Akt/eNOS pathway which regulates apoptosis of vascular endothelium caused by high glucose levels [13]. The above-mentioned mechanisms foster the development of CAD in T2DM.

It has been reported that elevated levels of low-density lipoprotein cholesterol (LDL-C) and decreased levels of high-density lipoprotein cholesterol (HDL-C) are vital risk factors for the increased risk of CAD in patients with T2DM [14, 15]. With apolipoprotein B (ApoB) as one of components, LDL-C is a cholesterol-rich lipoprotein that promotes the excessive lipid enter the arterial intima through pinocytosis and then participate in foam cell transformation and arterial plaque formation [16]. Apolipoprotein A1 (ApoA1) mainly exists in HDL-C. It plays an important role in balancing LDL-C function and promoting cholesterol’s reverse transport to the liver, followed by reducing LDL-C deposition in vascular walls and protecting against functional damage in islet beta cells [15, 17]. There was evidence that high ApoB/ApoA1 ratio may predict the severity of CAD [14]. Lp(a) has long been explored by many researchers for its novel function in cardiovascular diseases, and abnormal increase in Lp(a) has been seen as an established risk factor for CAD. It is genetically variable between individuals and is an atherogenic factor [18, 19]. However, unlike Lp(a), the ApoB/ApoA1 ratio has not been studied in detail in CAD patients. Given the increasingly important role of GDF-15 and lipoproteins in T2DM and CAD, this study aimed to evaluate and compare the ApoB/ApoA1 ratio and GDF-15 concentrations in T2DM patients with or without CAD and examine their ability to predict CAD.

## Methods

### Subjects

**Fig. 1:**
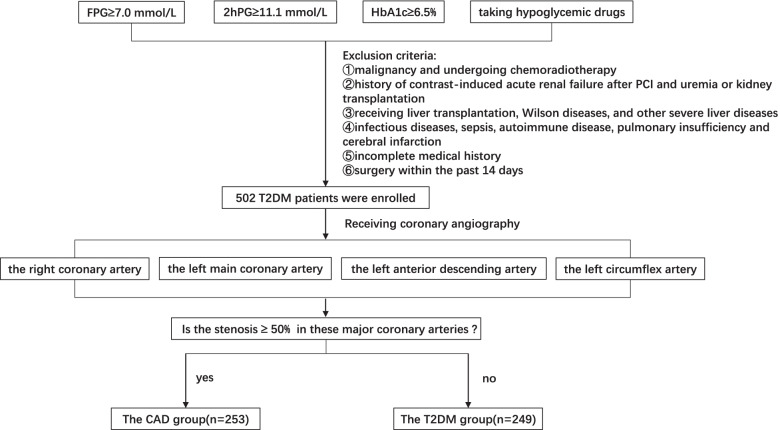
Diagram of patients selection. Abbreviations: FPG, fasting blood glucose; 2hPG, 2 h postprandial blood glucose; HbA1c, glycosylated hemoglobin; T2DM, type 2 diabetes mellitus; CAD, coronary artery disease

All participants in this study were recruited from the Department of Cardiovascular Medicine and Endocrinology, Renmin Hospital of Wuhan University. Past medical history, lifestyle, and drug use of all patients were obtained before their admission. T2DM patients with or without CAD were all eligible for the study and consecutive patients were also included. The following are exclusion criteria: (1) malignancy and undergoing chemoradiotherapy, (2) history of contrast-induced acute renal failure after PCI and uremia or kidney transplantation, (3) receiving liver transplantation or other severe liver diseases, (4) infectious diseases, sepsis, autoimmune disease, pulmonary insufficiency, and cerebral infarction, (5) incomplete basic information such as medication history and previous disease history, (6) history of surgery within the past 14 days. According to inclusion and exclusion criteria, 502 diabetic subjects were included from January 2021 to December 20 and were divided into either a T2DM group (*n* = 249) or CAD group (*n* = 253) (Fig. 1).

### Study definition

The diagnosis of T2DM was consistent with the standards issued by ADA [20]. Smokers and drinkers referred to those who had not quitted their smoking or drinking habits, respectively, from the past to the present. BMI was calculated as; “weight (kg) / height^2^(meters)”. Hypertension was defined by the WHO principles [21].

### Coronary angiography and diagnosis of CAD

coronary angiography was performed to diagnose CAD and the latter was defined as greater than 50% stenosis of the major coronary arteries [22]. Coronary arteriography was performed by experienced interventional doctors and the results then analyzed and admitted by at least two cardiologists. The clinical materials were obtained from corresponding physicians who were unaware of the research’s methodology and objective. A new diagnosis of CAD was not made during the research.

### Blood sampling and laboratory analysis

All patients fasted for at least 8 hours after admission. The blood sample was collected in tubes containing separating gel and EDTA-K2, and then centrifuged for serum collection, followed by storage until measurement. GDF-15 was quantified using a sandwich ELISA kit (R&D Systems, USA) with horseradish peroxidase involved in the color reaction. Hemolyzed specimens were strictly prohibited from measurement. Optical density was determined immediately in microplate reader (Autobio Instruments, PHOMO, Zhengzhou, Henan, China).

Leucocyte and its classification were counted on a Sysmex XN-20 (Kobe, Japan). HbA1c was determined in Trinity Biotech Premier Hb9210 kit (Kansas, USA). Total cholesterol (TC), triglycerides (TG), blood glucose (Glu), glycated albumin (GA), free fatty acids (FFA), HDL-C, LDL-C, ApoA1 and ApoB were measured on a Siemens automatic biochemical analyzer ADVIA 2400 (Erlangen, Germany). The CKD-EPICr formula recommended by the NKF-ASN Task Force was performed to compute the estimated glomerular filtration rate(eGFR) [23].

### Statistical analysis

All data were analyzed in SPSS 23.0 and R 3.5.2. BMI obeyed normal distribution and was therefore presented as mean ± standard deviation with difference determination using Students t-test. Other continuous variables belonged to non-normally distributed data and they were given as interquartile range, then analyzed with the Mann-Whitney test. Categorical data including insulin, metformin, and anti-hypertensive treatment, drinking, smoking, hypertension and gender were expressed as percentages then analyzed by means of the chi-square test. Logistic regression was conducted and then analyzed in SPSS 23 software to examine the relationship between GDF-15 or ApoB/A1 and the prevalence of CAD in T2DM patients. Finally, further analysis of the fully adjusted model was performed using restricted cubic spline analysis.

## Results

### Characteristic of subjects

The characteristics of 502 T2DM subjects were showed in Table 1. Diabetic duration, BMI, age, leukocyte, neutrophil, NLR, hs-CRP, 2hPG, GA, GA/ALB, GDF-15, AST, ApoB/ApoA1 ratio, Urea, Cr, FFA, and ApoB increased in the CAD group, while LYM, HDL-C, ApoA1 and eGFR were lower. The type, severity, and duration of CAD in the 253 patients were shown in Additional file (Table X1).

**Table 1:** Characteristic of T2DM patients with and without CAD

Characteristic	T2DM without CAD(*n* = 249)	T2DM with CAD(*n* = 253)	*p* value
Clinical variables			
Age (years)	51(42,59)	59(53,67)	< 0.001
Male (%)	54.15	48.19	0.182
Diabetic durations (years)	1(0,4)	3(0,6)	< 0.001
BMI	24.04 ± 0.11	24.93 ± 0.14	0.002
Medical history (n, %)			
Smoking (%)	20.95	18.07	0.432
Alcohol drinking (%)	14.22	13.65	0.853
Hypertension (%)	32.41	28.92	0.396
Medication (n, %)			
Insulin (%)	62.06	69.48	0.080
Metformin (%)	47.04	50.60	0.424
Anti-hypertension	28.85	23.69	0.189
Laboratory variables			
WBC (× 10^9^/L)	6.09(5.18,7.02)	7.31(5.95,8.64)	< 0.001
NEU (× 10^9^/L)	3.30(2.66,4.07)	4.53(3.02,6.07)	< 0.001
LYM (× 10^9^/L)	1.95(1.55,2.39)	1.73(1.28,2.15)	< 0.001
NLR ratio	1.66(1.30,2.15)	2.50(1.61,4.24)	< 0.001
hsCRP (mg/dl)	0.80(0.34,2.56)	1.69(0.64,3.74)	< 0.001
HbA1c (%)	8.5(6.8,10,4)	8.8(7.5,9.0)	0.334
FPG (mmol/L)	9.76(7.03,12.22)	9.04(7.52,11.45)	0.569
2hPG (mmol/L)	14.37(10.99,18.81)	18.31(13.22,21.82)	< 0.001
GA (g/L)	9.54(6.86, 12.30)	10.76(8.97,12.74)	< 0.001
GA/ALB (%)	0.234(0.174,0.299)	0.354(0.307,0.407)	< 0.001
ALT (U/L)	19(14,28)	21(15.29)	0.065
AST (U/L)	17(13,22)	21(16.27)	< 0.001
GGT (U/L)	27(18,32)	28(19,36)	0.968
Urea (mmol/L)	5.44(4.50,6.35)	5.88(4.75,7.08)	< 0.001
Cr (μmol/L)	64(57,72)	73(64,82)	< 0.001
UA (μmol/L)	350(288,423)	342(286,423)	0.614
eGFR (ml/min/1.73m^2^)	108.35(98.77,116.57)	97.63(90.48,106.22)	< 0.001
FFA (mmol/L)	0.31(0.20,0.45)	0.54(0.34,0.91)	< 0.001
TC (mmol/L)	4.56(3.86,5.17)	4.47(3.79,5.31)	0.909
TG (mmol/L)	1.60(1.12,2.87)	1.56(1.02,2.30)	0.135
HDL-C (mmol/L)	0.96(0.82,1.17)	0.89(0.77,1.07)	0.002
LDL-C (mmol/L)	2.53(1.91,3.22)	2.40(1.68,3.23)	0.347
Apo A1 (g/L)	1.22(1.09,1.35)	1.17(1.05,1.28)	0.003
Apo B (g/L)	0.82(0.69,0.95)	0.90(0.74,1.02)	< 0.001
Apo B/Apo A1 ratio	0.68(0.55,0.81)	0.78(0.60,0.91)	0.001
GDF-15 (pg/dl)	7.88(5.92,10.11)	13.84(9.43,18.41)	< 0.001

All data are given in the form of mean ± standard deviation, median (25th percentile-75th percentile) and percentage

Abbreviations: *BMI* body mass index, *WBC* white blood cell, *NEU* neutrophil, *LYM* lymphocyte, *NLR* neutrophil to lymphocyte ratios, *hs-CRP* high-sensitivity C-reactive protein, *HbA1c* glycated hemoglobin A1c, *FPG* fasting plasma glucose, *2hPG* 2 h-plasma glucose, *GA* glycated albumin, *GA/ALB* glycated albumin ratio, *ALT* alanine aminotransferase, *AST* aspartate aminotransferase, *GGT* γ-glutamyl transpeptidase, *Cr* creatinine, *UA* uric acid, *eGFR* estimated glomerular filtration rate (mL/min/1.73 m^2^), *FFA* free fatty acid, *TC* total cholesterol, *TG* triglyceride, *HDL-c* high-density lipoprotein cholesterol, *LDL-c* low-density lipoprotein cholesterol, *Apo A1* apolipoproteins A1, *Apo B* apolipoproteins B, *GDF15* growth differentiation factor 15

### Logistic analysis of the prevalence of CAD in T2DM patients and serum GDF-15 concentrations and ApoB/ApoA1 ratio

To further analyze the correlation between CAD and GDF-15 or ApoB/ApoA1 ratio in the T2DM patients, the data were divided into quartiles of GDF-15 or ApoB/ApoA1 ratio, taking the first quartiles as the reference to calculate the odds ratio (OR) for CAD, and the results were shown in Tabless 2 and 3. GDF-15 and ApoB/ApoA1 ratio in T2DM patients presented a positive association with the prevalence of CAD in the Crude model (*p* < 0.01). After adjustment for insulin, metformin, and antihypertensive treatment, drinking, smoking, BMI, hypertension, diabetic duration, age, gender in Model 1, the results remained consistent with the Crude model (*p* < 0.01). The difference also remained statistically significant after further control of Urea, Cr, WBC, NEU, LYM, NLR, hs-CRP, UA, eGFR, HbA1c, FPG, 2hPG, GA, GA/ALB, FFA, LDL-C, HDL-C TC, TG, ALT, AST and GGT. The fully adjusted OR in Model 2 was 11.514(95%CI:4.586,28.909) for quartile 4 of circulating GDF-15 concentrations (the highest) versus quartile 1 (the lowest), and was 2.388(95%CI:1.891,10.245) for quartile 4 of ApoB/ApoA1 (the highest) versus quartile 1 (the lowest).

**Table 2:** Association of coronary heart diseases with serum GDF-15 in T2DM patients

GDF-15 quartiles	n	Concentration range	OR (95%CI)		
			Crude	Model 1	Model 2
Quartile 1(low)	125	≤7.030	Reference	Reference	Reference
Quartile 2	126	7.030–9.935	2.426(1.345,4.373)	2.292(1.218,4.315)	2.417(0.995,5.868)
Quartile 3	126	9.935–15.177	10.842(5.967,19.700)	9.442(5.007,17.805)	5.250(2.068,13.328)
Quartile 4(high)	125	≥15.177	15.498(8.337,28.810)	9.502(4.916,18.366)	11.514(4.586,28.909)
β			−0.990	−0.838	−0.656
SE			0.100	0.106	0.146
p value			< 0.001	< 0.001	< 0.001

Logistic regression was used to examine the associations between serum levels of GDF-15 and coronary artery diseases in T2DM patients. Serum GDF-15 was divided into quartiles (quartile 4: ≥75th, quartile 3: 50–75th, quartile 2: 25–50th, quartile 1: < 25th percentile)

Crude: no adjustment

Model 1: adjusted for age, gender, diabetic durations, BMI, alcohol drinking, smoking, hypertension, insulin, metformin and anti-hypertension treatments

Model 2: adjusted for the same variables as Model 1 as well as WBC, NEU, LYM, NLR, hs-CRP, HbA1c, FPG, 2hPG, GA, GA/ALB, ALT, AST, GGT, Urea, Cr, UA, eGFR, FFA, TC, TG, HDL-C, LDL-C, ApoB, ApoA1 and ApoB/ApoA1

**Table 3:** Association of coronary heart diseases with serum Apo B/Apo A1 ratios in T2DM patients

ApoB/ApoA1 quartiles	n	Ratio range	OR (95%CI)		
			Crude	Model 1	Model 2
Quartile 1(low)	122	≤0.5772	Reference	Reference	Reference
Quartile 2	130	0.5772–0.7358	1.171(1.026,2.852)	1.784(1.007,3.161)	1.310(0.580,2.961)
Quartile 3	124	0.7358–0.8716	2.850(1.707,4.759)	3.237(1.821,5.754)	2.342(1.291,7.402)
Quartile 4(high)	125	≥0.8716	2.867(1.714,4.798)	3.327(1.864,5.940)	2.388(1.891,10.245)
β			−0.366	−0.286	−0.351
SE			0.083	0.091	0.135
p value			< 0.001	< 0.002	0.001

Logistic regression was used to examine the associations between serum levels of GDF-15 and coronary artery diseases in T2DM patients. Serum GDF-15 was divided into quartiles (quartile 4: ≥75th, quartile 3: 50–75th, quartile 2: 25–50th, quartile 1: < 25th percentile)

Crude: no adjustment

Model 1: adjusted for age, gender, diabetic durations, BMI, alcohol drinking, smoking, hypertension, insulin, metformin and anti-hypertension treatments

Model 2: adjusted for the same variables as Model 1 as well as GDF-15, WBC, NEU, LYM, NLR, hs-CRP, HbA1c, FPG, 2hPG, GA, GA/ALB, ALT, AST, GGT, Urea, Cr, UA, eGFR, FFA, TC, TG, HDL-C, and LDL-C

### Restricted cubic spline analysis

**Fig. 2:**
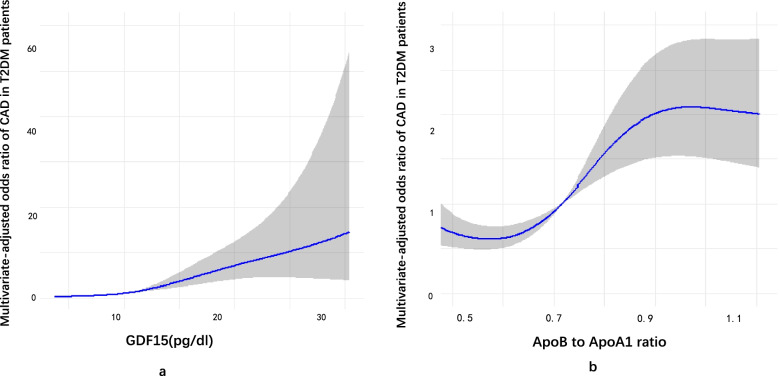
Restricted cubic spline model of the odds ratios of CAD with serum GDF-15 and ApoB to ApoA1 ratio in T2DM patients. The dashed lines represent the 95% confidence intervals. GDF-15: serum growth differentiation factor 15; Apo B, apolipoproteins B; Apo A1, apolipoproteins A1. Both serum GDF-15 levels and ApoB/ApoA1 ratio were positively correlated with CAD

Figure 2a and b indicated the association between the prevalence of CAD in T2DM population and GDF-15 and ApoB/ApoA1 ratio. The grey area in these graphs showed the 95% confidence interval. The analysis was adjusted for insulin, metformin, anti-hypertensive treatment, drinking, smoking, BMI, hypertension, diabetic duration, age, gender, ALT, AST, GGT, WBC, NEU, LYM, NLR, hs-CRP, FFA, TC, TG, HDL-C, LDL-C, Urea, Cr, HbA1c, FPG, 2hPG, GA, GA/ALB, UA and eGFR. The results indicated that serum GDF-15 or ApoB/ApoA1 ratio correlated positively with the prevalence of CAD.

## Discussion

To the best of our knowledge, this is the first study to investigate the association between GDF-15 and ApoB/ApoA1 ratio and the prevalence of CAD in T2DM patients.

CAD is one of terrible comorbidities of T2DM, as well as the leading cause of death in T2DM patients [2]. Investigations have indicated that the risk of CAD in diabetic men is up to 2 times higher than that in normal men, while CAD in women with diabetes is appropriately 3 times higher than that of normal women [24]. As an inflammatory factor, serum GDF-15 is up-regulated when inflammatory damage occurs in vascular endothelial cells, pancreatic islet β cells, and cardiomyocytes, a process possibly involving TGFβRII and IL-6. The formation of atherosclerotic plaque is essentially the inflammation of vascular wall, and GDF-15 involved in this process [10, 12, 25, 26]. Consistent with previous reports, findings in this research showed that subjects with diabetes and CAD had higher levels of WBC, NEU, NLR, hs-CRP and GDF-15 than those with T2DM alone, indicating a more severe inflammatory state. GDF-15 is secreted from endothelial cells under high glucose conditions through ROS- and p53-dependent pathways, and acts in the occurrence and development of CAD [13]. Furthermore, another study pointed that the increase in circulating GDF-15 levels predicted the severity of coronary atherosclerosis and CAD, as well as raised the mortality of adverse cardiovascular events [27]. These findings suggest that the activation of hyperglycaemia-endothelial-GDF15-inflammation pathway may lead to myocardial damage. However, it should be noted that there is insufficient evidence supporting that controlling GDF-15 concentrations effectively leads to low incidence rate of CAD [28].

Results in this study showed that T2DM patients with CAD had significantly higher GDF-15 levels than those with T2DM only. Being a novel biomarker, however, not all studies showed that circulating GDF-15 levels increased in diabetics [25, 28]. Recent literatures found that GDF-15 predicted the ventricular remodeling in healthy individuals and diabetic patients [26, 29, 30]. In the heart, GDF-15 might activate the ALK receptor and phosphorylate smad protein, and inhibit the NF-κB related pathway and EGFR trans-activation to contribute to cardiac inflammation and ventricular remodeling [31]. In macrophages, GDF-15 induces the expression of ABCA1 by triggering the PI3-K related pathway, and then indirectly results in atherosclerosis [32]. Furthermore, the up-regulated GDF-15 also involves in the damage on endothelial cell mediated by the increased glucose levels via attenuating NF-1 and activating the PI3K/AKT/eNOS signaling pathway [33]. Despite the mechanism by which GDF-15 acts remains unclear, we speculate that it is the existence of above-mentioned signaling pathways or signaling molecules that makes GDF-15 a potential inflammatory factor connecting T2DM with CAD.

Both T2DM and CAD exist lipid metabolism disorders, with a large number of studies having shown that dyslipidemia acts as a crucial part in the occurrence of CAD in T2DM patients [34, 35]. A retrospective study conducted in Romanian T2DM populations found that abnormal blood lipids were an important cause of CAD and up to 91.48% of subjects had both CAD and a history of receiving lipid-lowering drugs such as atorvastatin [34]. Another observational study in Swedes with T2DM indicated that serum non-HDL-C to HDL-C ratio might predict CAD [36]. According to existing literature, it can be assumed that dyslipidemia acts as a vital role in T2DM and CAD.

ApoB/ApoA1 ratio is elevated in diabetics with CAD in this study, but the mechanism is unclear. Voluminous literature revealed that the development of CAD in diabetic patients was a long-term process involving increased LDL-C levels and decreased HDL-C levels, accompanied by the continuous formation of subintimal foam cells [36, 37]. A multivariate Mendelian randomization study showed that the ApoB in LDL may be a major feature of the serum lipid profile and etiology of CAD [16, 38]. Thomas et al. found that administration of ApoB synthesis inhibitors significantly reduced the risk of CAD [39], indicating the potential value of ApoB. ApoA1 is a constituent of HDL-C and participates in hepatic cholesterol metabolism. Another study from a large-scaled population indicated that the combination of HDL-C and ApoA1 predicted the occurrence of CAD, as well as presenting a close association between ApoA1 and the risk factors of CAD, including high BMI, CRP, and ApoB [40]. Serum ApoB/ApoA1 ratio was also reportedly associated with first myocardial infarction [41]. These findings suggest that ApoB/ApoA1 ratio might act as a potential marker, providing novel perspectives for explaining the associations between CAD and T2DM.

### Study strength and limitations

The current research indicated, for the first time, that the ApoB/ApoA1 ratio and concentrations of serum GDF-15 were predictive indicators of CAD in Chinese patients with T2DM, independent of potential risk factors such as hyperglycemia, diabetic duration, hypertension and age. Therefore, dynamically monitoring variations in the ApoB/ApoA1 ratio and GDF-15 concentration has high value for clinical management of T2DM.

However, several limitations in this study should be considered. First, the serum samples were collected from Chinese, which means the generalizability of the study findings to different regions or ethnicities requires further verification. In addition, statistical power in this research was limited by the small sample size. Third, despite adjustments for medication and other confounders, it was not possible to rule out potential elements such as the effect of other drugs on GDF-15, ApoB and ApoA1 that were not recorded in this study. Fourth, follow-up data applicable to long-term prognosis was missing in the patients with T2DM.

## Conclusion

All in all, circulating GDF-15 levels and serum ApoB/ApoA1 ratio in T2DM patients with CAD were higher than those who with T2DM only. Serum GDF-15 levels and the ApoB/ApoA1 ratio may therefore be helpful in T2DM patients for predicting CAD and preventing adverse cardiovascular events.

## Supplementary Information

**Additional file 1: Table X1.** Characteristic of 253 CAD patients

## Data Availability

The datasets generated during and analyzed during the current study are not publicly available due to privacy or ethical restrictions but are available from the corresponding author on reasonable request.

## Abbreviations

GDF-15: Growth differentiation factor 15
ApoB: Apolipoprotein B
ApoA1: Apolipoprotein A1
CAD: Coronary artery disease
T2DM: Type 2 diabetes mellitus
DM: Diabetes mellitus
BMI: Body mass index
WBC: White blood cell
NEU: Neutrophil
LYM: Lymphocyte
NLR: Neutrophil to lymphocyte ratios
hs-CRP: High-sensitivity C-reactive protein
HbA1c: Glycated hemoglobin A1c
FPG: Fasting plasma glucose
2hPG: 2 h-plasma glucose
GA: Glycated albumin
GA/ALB: Glycated albumin ratio
ALT: Alanine aminotransferase
AST: Aspartate aminotransferase
GGT: γ-glutamyl transpeptidase
Cr: Creatinine
UA: Uric acid
eGFR: Estimated glomerular filtration rate
FFA: Free fatty acid
TC: Total cholesterol
TG: Triglyceride
HDL-c: High-density lipoprotein cholesterol
LDL-c: Low-density lipoprotein cholesterol
ROC: Receiver operating curve
