# Supplementary material for: Circulating growth differentiation factor 15 levels and apolipoprotein B to apolipoprotein A1 ratio in coronary artery disease patients with type 2 diabetes mellitus
Source: Lipids Health Dis. 2022 Jul 16;21:59. doi: 10.1186/s12944-022-01667-1 (PMC9287968; doi:10.1186/s12944-022-01667-1)
Supplement: Supplementary file 1 — Additional file 1: Table X1. Characteristic of 253 CAD patients [file 12944_2022_1667_MOESM1_ESM.docx]

**Table X1 .** Characteristic of 253 CAD patients

| **Type** | | **Severity (%)** | **CAD duration(years)** |
| --- | --- | --- | --- |
| stable angina pectoris(n=65) | | CCS I, 4.74% | 0 (0, 3) |
|  |  | CCS II, 11.07% |  |
|  |  | CCS III, 7.51% |  |
|  |  | CCS IV, 2.37% |  |
| ACS | UAP(n=95) | Braunwald I, 19.37% | 0 (0, 1) |
|  |  | Braunwald II, 13.04% |  |
|  |  | Braunwald III, 5.14% |  |
|  | AMI(n=93) | Killip I, 23.72% | 0 (0, 2) |
|  |  | Killip II, 9.48% |  |
|  |  | Killip III, 1.97% |  |
|  |  | Killip IV, 1.58% |  |
| Total(n=253) | |  |  |

**Note:** durations of CAD are presented as the 25th percentile-75th percentile. The severity of stable angina pectoris is expressed by the Canadian Cardiovascular Association classification (from I to IV). The severity of UAP and AMI was expressed by Braunwald classification (from I to III) and Killips classification (from I to IV), respectively.

**Abbreviation:** ACS, acute coronary syndrome; UAP, unstable angina pectoris; AMI, acute myocardial infarction; CCS, Canadian Cardiovascular Society
